# Cochlear Damages Caused by Vibration Exposure

**DOI:** 10.5812/ircmj.5369

**Published:** 2013-09-05

**Authors:** Seyyed Ali Moussavi Najarkola, Ali Khavanin, Ramazan Mirzaei, Mojdeh Salehnia, Ahad Muhammadnejad

**Affiliations:** 1 Department of Occupational Hygiene, Collage of Health, Shahid Beheshti University of Medical Sciences, Tehran, IR Iran; 2 Department of Occupational Health, School of Medical Sciences, Tarbiat Modares University, Tehran, IR Iran; 3 Department of Occupational Health, Health Promotion Research Center, Zahedan University of Medical Sciences,Zahedan, IR Iran; 4 Department of Anatomical Sciences, School of Medical Sciences, Tarbiat Modares University, Tehran, IR Iran; 5 Cancer Research Center, Iran Cancer Institute, Tehran University of Medical Sciences, Tehran, IR Iran

**Keywords:** Inner Auditory Hair Cell, Cochlear Hearing Loss, Histological Technique

## Abstract

**Background:**

Many industrial devices have an excessive vibration which can affect human body systems. The effect of vibration on cochlear histology has been as a debatable problem in occupational health and medicine.

**Objectives:**

Due to limitation present in human studies, the research was conducted to survey the influence of vibration on cochlear histology in an animal model.

**Materials and Methods:**

Twelve albino rabbits were experimented as: Vibration group (n = 6; exposed to 1.0 m.s^-2^ r.m.s vertical whole-body vibration at 4 - 8 Hz for 8 hours per day during 5 consecutive days) versus Control group (n = 6; the same rabbits without vibration exposure). After finishing the exposure scenario, all rabbits were killed by CO_2_ inhalation; their cochleae were extracted and fixed in 10% formaldehyde for 48 hours, decalcified by 10% nitric acid for 24 hours. Specimens were dehydrated, embedded, sectioned 5 µm thick and stained with Hematoxylin and Eosin for light microscopy observations.

**Results:**

Severely hydropic degenerated and vacuolated inner hair cells (IHCs) were observed in vibration group compared to the control group. Inter and intracellular edema was appeared in supporting cells (SC). Nuclei of outer hair cells (OHCs) seemed to be pyknotic. Slightly thickened basilar membrane (BM) was probably implied to inter cellular edematous. Tectorial Membrane (TM) was not affected pathologically.

**Conclusions:**

Whole-body vibration could cause cochlear damages in male rabbits, though vibration-induced auditory functional effects might be resulted as subsequent outcome of prolonged high level vibration exposures.

## 1. Background

Many industrial devices have an excessive whole-body vibration (WBV) affecting human body systems. WBV is caused by vibration transmitted through the seat or the feet by workplace machines and vehicles ranged from 0.5 to 80 Hz (1). Along with musculoskeletal problems, exposure to occupational WBV also presents a health risk to the psychomotor, physiological, and psychological systems of the body (2). High levels WBV Exposure can present risks to health and safety and are reported to cause or aggravate back injuries (3). During transmitting WBV to the body, the effect of the vibration can be amplified by factors such as body posture, type of seating and frequency of the vibration. WBV can agitate the body to the point of causing micro fractures in the vertebrae, disc protrusion, nerve damage and acute lower back pain (4).

Effect of vibration on cochlea has been a debatable subject during the past decades (5). Little information is available about chronic effects of vibration on hearing, since human responses depend on vibration frequency, magnitude and duration, body posture, and subject susceptibility (6). Most reports are referred to the hearing function measures (7-14). Hamernik et al. (1980) showed that vibration may induce hearing loss or cochlear damages (10). While, Yokoyama et al. (1973) showed no significant change in threshold sensitivity after exposure to vibration alone (14). Okada et al. (1971) reported vibration-induced temporary threshold shift (TTS) due to resonance frequency of human body (12). Hamernik et al. (1981) found no vibration effect on hearing threshold (9). While, Hamernik et al. (1989) showed altered shape of permanent threshold shift (PTS) audiogram due to vibration exposure (8). Bochnia et al. (2005) professed low and medium frequency-vibration damages to the inner ear structures (7). Soliman et al. (2003) asserted that WBV exposure enhanced DPOAEs amplitudes and signal to noise ratios (SNR) (13). Moussavi-Najarkola et al. (2012) showed larger DPOAE amplitudes (Adp) at mid to high frequencies in vertical WBV-exposed rabbits (11). But, only a few reports are present regarding the influence of vibration on cochlea. Temkin (1993) showed vibration synergistic effect on noise-induced cochlear damage in mouse (15). Hamernik et al. (1989) found that vibration exposure induce evident outer hair cell (OHCs) losses (8). While, Bochnia et al. (2005) asserted vibration-induced damages in the apex of the outer hair cells' third row towards the modiolus (7). Soliman et al. (2003) revealed more damage to inner hair cells (IHCs) than OHCs in WBV-exposed guinea pigs (13).

## 2. Objectives

Due to equivocal role of WBV on cochlear damage in the recent decades, this study was conducted to examine the histological cochlear changes following prolonged WBV exposure at realistic industrial levels on animal model.

## 3. Materials and Methods

### 3.1. Model

**Figure 1.**
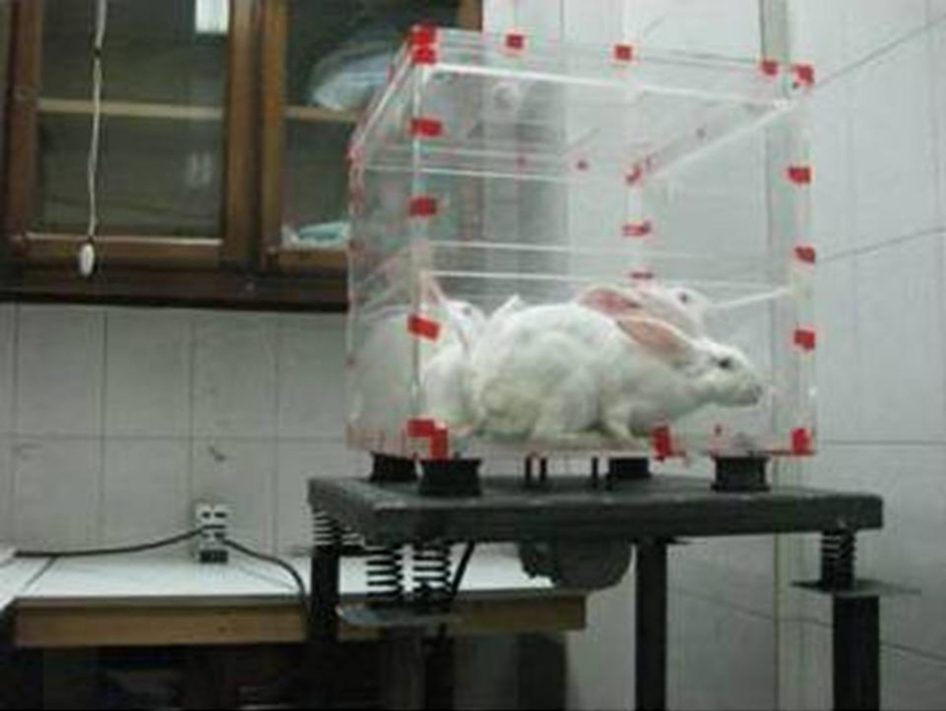
Cross-sectional View of Transparent Poly Carbonate Plexiglas Vibration Chamber Assembled on a Vibrating Platform Consisted of a Three-Phase Body Vibrator (Model ITAL VIBREH; M3/65; Italia) for Creating Vibration and an Inverter (Model LG; 0.37 KW IG5A-4; Korea).

Twelve randomly sampled albino rabbits were experimented as two groups: Control group (n = 6; not exposed to vibration) versus Vibration group (n = 6; exposed to defined whole body vibration). Two-sample Kolmogorov-Smirnov analysis was used to evaluate the normality of gathered data. Power analysis was also used to compute the minimum sample size needed to achieve a significant pathologic result in each group (H_0 _: = 6). All rabbits underwent "Distortion Product Otoacoustic Emissions (DPOAEs)" for cochlear hair cells health and "Tympanometry" for middle ear health before the experiments. The experimental protocol was: baseline audiometry (for cochlear health screening on day 0), rest periods (3 days; days 1 through 3), exposure periods (only for vibration rabbits; vibration exposure on days 4 through 8), rest period (3 days; days 9 through 11), and histological examination (on day 11). Vibration rabbits were exposed to 1.0 m.s^-2 ^r.m.s vertical whole-body vibration (WBV) at 4 - 8 Hz for 8 hours per day during 5 consecutive days (based on ISO-2631) by putting them onto a 50×50×50 cm transparent poly-carbonated Plexiglas exposure chamber inserted on a vibrating platform (as shown in Figure 1). Vibration chamber was consisted of three components including: mass (total mass of the chamber, 8 spring shock absorbers, metal plate with the dimensions of 50×50 cm, mounts, rabbits weights, and 4 compressed plastic shock absorbers were equal to about 45 kg), stiffness (spring and shock absorbers), and damping. Vibrating platform was also consisted of a three-phase body vibrator (Model ITAL VIBREH; M3/65; Italy) and an inverter (Model LG; 0.37 KW IG5A-4; Korea). Vibration chamber was designed so that 10 complete air changes were provided through (16) opening with 5 cm diameter in four lateral faces as well as 2 windows with 19×11.5 cm dimensions in the ceiling. Environmental background noise was less than 5 dB. Minimum requirements of the Guide for the Care and Use of Laboratory Animals (ILAR 1985) (16) were absolutely provided. The "General Principles of Helsinki Law related to Laboratory Animal" (OLAW 2002) (17) was completely observed. Animal house temperature was maintained between 20°C and 22°C, relative humidity was maintained between 30% and 70%, and ventilation rate was 10 complete air changes per hour with 100% fresh air. Facilities maintained the light cycles of 12 hour light and 12 hour dark. Minimum cage size was around 0.14 m^2 ^floor area per rabbit up to 2000 ± 200 grams. Feed and water systems were clean and designed so that they could not become easily contaminated. Rabbits were provided ad libitum with a plentiful supply of fresh and clean water. Rabbits were fed to free access to Purina (ingredients of raw protein, raw fiber, raw fat, metabolic energy, calcium, phosphorus, salt, and humidity with definite percentage). 

### 3.2. Histological examination

At the end of experimental scenario, rabbits were anaesthetized through carbon dioxide inhalation, decapitated, and cochleae were removed. Extracted cochleae were fixed in 10% formaldehyde solution for 48 hours and decalcified with 10% nitric acid for 24 hours. Specimens were dehydrated, embedded, sectioned 5 μm thick and stained with Hematoxylin and Eosin (H&E) for light microscope (LM) examination. First, control animals' slides were pathologically examined regarding cell size, intercellular spaces, and relative cell count as well as cellular polarity degree in inner hair cells (IHCs), outer hair cells (OHCs), supporting cells (SC), basilar membrane (BM), and tectorial membrane (TM). Then, control slides were standardized, and a score 0 was allocated to any parameter of them as criteria. Then, vibration rabbits' slides were compared to these criteria in the blind situation and scores -2, -1, 0, +1, and +2 were belonged to any morphological changes. Thereby, any proliferation, atrophy, edema and cell injury were quantified.

## 4. Results

**Figure 2.**
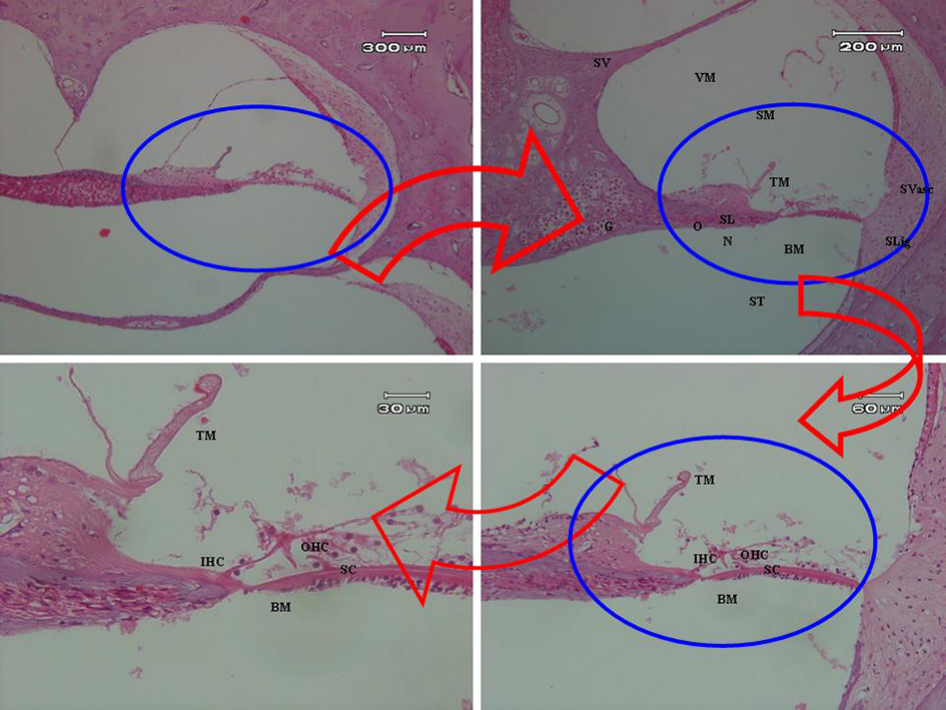
The Organ of Corti of Control Rabbits Showed Normal Cochlear Inner Hair Cells (IHCs), Outer Hair Cells (OHCs), Supporting Cells (SC), Basilar Membrane (BM), and Tectorial Membrane (TM).

Gathered data was confirmed to have normal distribution in both the control and vibration groups (C.I. = 0.95; Z = 262; P < 0.005) using KS. The sample size of the study design was enough to get significant pathologic results at an effect size of 80.5% of the cases in each group. All prepared slides were examined under light microscopy, and then observed data related to histopathological changes were generalized as representative pathological changes of that group. Normal cochlear inner hair cells (IHCs), outer hair cells (OHCs), supporting cells (SC), and tectorial membrane (TM) were shown in all slides of control rabbits (as shown in Figure 2). No abnormal changes were observed in control slides examination. 

**Figure 3.**
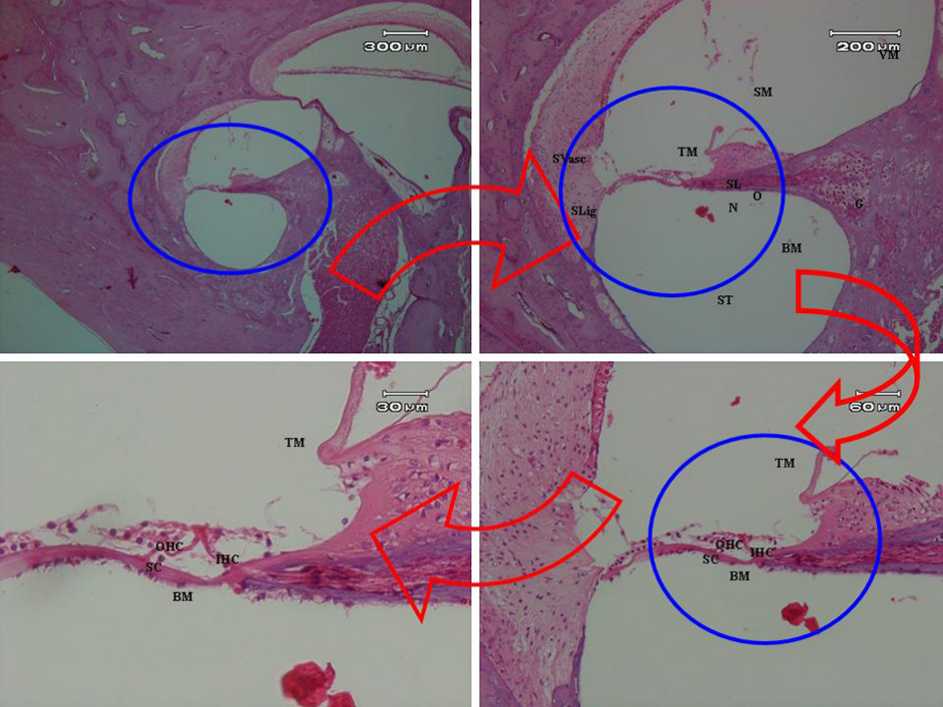
The organ of corti of rabbit exposed to vibration revealed severely hydropic degenerated and vacuolated IHCs, inter and intracellular edematous SC, euchromatin nuclei of OHCs, slightly thickened BM due to intercellular edematous.

While, very highly hydropic degeneration and severely vacuolation of inner hair cells (IHCs) were observed obviously in slides of vibration group (See Figure 3), inter and intracellular edemas found to be clear in inner hair cells (IHCs) and supporting cells (SC). Nuclei of outer hair cells (OHCs) appeared to be lightly euchromatin, so that it was observed to be more pallid in H & D staining compared to the control group, but this status was not extended in all slides of this group. It is likely that increased thickened basilar membrane (BM) is related to intercellular edema. No pathologic changes were found in tectorial membrane (TM).

Some studies believe that the vibration-induced hearing shifts measured through pure tone audiometry (PTA), auditory brainstem responses (ABR), and distortion product otoacoustic emissions (DPOAEs) are resulted from the cochlear damages happened on the auditory system.7-14 Consistent with the present study, Okada et al. (1971) cited that temporary threshold shift (TTS) occurred after both 20 and 60 min of exposure to the vibration of acceleration 500 cm/sec^2^ and frequency 5 Hz, which is regarded to the resonance frequency of human body.9 Also, Hamernik et al. (1989) professed that only stronger vibration exposure conditions (30-Hz, 3 g r.m.s) can alter the dependent measures of hearing and can alter the shape of the permanent threshold shift (PTS) audiogram (8). Hamernik et al. (1980) reported that vibration may induce or produce hearing loss or cochlear damages. Based on their opinion, although low frequencies (< 100 Hz) are relatively ineffective in initiating an auditory percept, they can vibrate the membranous labyrinth if levels are high enough (10). Bochnia et al. (2005) asserted that vibration-induced damages done to the inner ear structures may cause a worsening of hearing there, especially in the low and medium frequencies (7). Furthermore, Soliman et al. (2003) reported that the exposure to vibration only led to enhancement of both DPOAEs amplitudes and signal to noise ratios (SNR) (13). As well, Moussavi-Najarkola et al. (2012) showed that 1.0 m.s^-2^ r.m.s whole-body vibration (WBV) in Z-axis at 4-8 Hz frequency for 8 hours per day during 5 consecutive days has been associated with enhanced larger DPOAE amplitudes (A_dp_) at mid to high frequencies compared to control rabbits (11). Contradictory to this study, little other studies did not report any hearing function measurements resulted from cochlear damages. Yokoyama et al. (1973), for example, showed that there was no significant change in the threshold sensitivity after exposure to vibration alone (14). Hamernik et al. (1981) showed that vibration alone had essentially no effect on threshold (9).

Long-term exposure to excessive whole-body vibration can lead to pathologically damage to the cochlear inner hair cells, outer hair cells, supporting cells, and basilar membrane. Inner hair cells had more affection than other sections of cochlea, spreading slowly to outer hair cells. So that, most studies showed that this affection can render as shifting the shape of PTS audiograms due to activation of the outer hair cells activity by vibrating third row of outer hair cells. Therefore, most studies showed that whole-body vibration can change the shape of hearing audiograms that did not mean as the vibration-induced hearing loss.

Whole-body vibration only, in realistic levels present in industrial workplaces, was found to cause cochlear damages in male albino rabbits experimented. This research refuses the predominating current opinion that vibration exerts nontraumatic or only a weakly traumatic effect on cochlea, though vibration-induced auditory functional effects may be occurred as subsequent outcome after cochlear damage resulted from long-term exposure to high level vibrations. Because vibration usually accompanies noise in industrial settings, the harmfulness effects of vibration in aggravating noise effects on auditory organs must largely be considered in future studies.

## 5. Discussion

Prolonged exposure to whole-body vibration in rabbits at realistic levels typically found in industrial settings leads to extreme vacuolation, severe hydropic degeneration, and inter- and intracellular edemas of inner hair cells (IHCs). Supporting cells (SC) were also suffered from inter- and intracellular edema. Nuclei of outer hair cells (OHCs) were sustained somewhat euchromatin. Thickness of basilar membrane (BM) was increasingly enhanced owing to intercellular edema. While no significant change was pathologically occurred in tectorial membrane (TM).

Some studies wholly revealed the common point that inner hair cells were mostly affected in whole-body vibration exposure at realistic levels presented in industrial settings (7-14). In consistent with this study, Temkin (1993) reported that whole-body vibration affects inner hair cells and can exacerbate the noise-induced cochlear outer hair cells damage on the experimented mouse (15). Hamernik et al. (1989) described that the histological change was evident primarily in the extent of the outer hair cell losses only for a 30-Hz, 3 g r.m.s vibration exposure condition and the more severely vibration exposure condition could initially induce the outer hair cell losses and the shape of permanent threshold shift audiograms in chinchillas(8). Soliman et al. (2003) also reported that the whole-body vibration exposure can cause more damage to the inner hair cells than the outer hair cells in guinea pigs, so that normal outer hair cells, severely vacuolation inner hair cells and edematous and vacuolated supporting cells were clearly found in vibration exposed animals (13). Consistently, Bochnia et al. (2005) asserted that vibration-induced changes were seen in all the examined inner ear in guinea gigs (that is, inner ear was mainly affected in whole-body vibration exposure), and hair-cell damage was more often seen in the region of the apex, spreading gradually in the direction of the base and from the circumference (the third row of the outer hair cells, OHCs) to the modiolus (7). Most of the mentioned reports have found that whole-body vibration had an effect on inner hair cells with normal outer hair cells (7, 8, 11, 13, 15). Furthermore, loss of afferent input (through inner hair cells) can reduce the activity in the efferent olivocochlear bundle (9, 13). After recovery from vibration exposure, vacuolation of inner hair cells was gradually disappeared (9, 13). This led to the return of olivocochlear bundle activity, with normalization of outer hair cells activity (4, 13).
